# Reversible Bronchiectasis in COVID-19 Survivors With Acute Respiratory Distress Syndrome: Pseudobronchiectasis

**DOI:** 10.3389/fmed.2021.739857

**Published:** 2021-11-30

**Authors:** Qiongjie Hu, Yiwen Liu, Chong Chen, Ziyan Sun, Yujin Wang, Min Xiang, Hanxiong Guan, Liming Xia

**Affiliations:** Department of Radiology, Tongji Hospital, Tongji Medical College, Huazhong University of Science and Technology, Wuhan, China

**Keywords:** reversible bronchiectasis, COVID-19, pneumonia, acute respiratory distress syndrome, follow-up

## Abstract

To retrospectively analyze whether traction bronchiectasis was reversible in coronavirus disease 2019 (COVID-19) survivors with acute respiratory distress syndrome (ARDS), and whether computed tomography (CT) findings were associated with the reversibility, 41 COVID-19 survivors with ARDS were followed-up for more than 4 months. Demographics, clinical data, and all chest CT images were collected. The follow-up CT images were compared with the previous CT scans. There were 28 (68%) patients with traction bronchiectasis (Group I) and 13 (32%) patients without traction bronchiectasis (Group II) on CT images. Traction bronchiectasis disappeared completely in 21 of the 28 (75%) patients (Group IA), but did not completely disappear in seven of the 28 (25%) patients (Group IB). In the second week after onset, the evaluation score on CT images in Group I was significantly higher than that in Group II (*p* = 0.001). The proportion of reticulation on the last CT images in Group IB was found higher than that in Group IA (p < 0.05). COVID-19 survivors with ARDS might develop traction bronchiectasis, which can be absorbed completely in most patients. Traction bronchiectasis in a few patients did not disappear completely, but bronchiectasis was significantly relieved. The long-term follow-up is necessary to further assess whether traction bronchiectasis represents irreversible fibrosis.

## Introduction

Coronavirus disease 2019 (COVID-19) caused by SARS-CoV-2 has become a global pandemic (1). About 80% of the COVID-19 patients have mild infection, and few patients may rapidly progress to acute respiratory distress syndrome (ARDS) (2, 3). The elderly patients and those with comorbidities are at the greatest risk of death, which may be associated with ARDS (4). The typical computed tomography (CT) feature of ARDS in the acute phase is the opacification that demonstrates an anterio-posterior density gradient within the lungs (5). In the late phase, the reticulation and ground-glass opacity (GGO) in the anterior part of lungs are the more typical CT findings (6, 7). When the patients with COVID-19 pneumonia progress to ARDS, CT images show expanded lung involvement, increased density, and consolidation (8, 9).

Traction bronchiectasis appears as irregular bronchiectasis on CT images, which is a common radiological characteristic of patients with ARDS in the acute phase (10, 11). It is also present in patients with COVID-19 pneumonia (8, 12, 13). Ambrosetti et al. (14) reported that two cases of COVID-19 pneumonia presented bronchiectasis progressing to ARDS. Our previous study suggested that patients with COVID-19 pneumonia had traction bronchiectasis in the acute phase of ARDS (15). Furthermore, traction bronchiectasis may affect the prognosis and increase the risk of death (16), and it is generally considered as the evidence of fibrosis (17, 18). However, recent studies have shown that bronchiectasis is reversible in patients with severe infection and inflammation, thus it is also known as pseudobronchiectasis (19–26). At present, reversible bronchiectasis in patients with COVID-19 pneumonia remains unclear (27). Therefore, this study aimed to explore whether traction bronchiectasis was reversible in COVID-19 pneumonia survivors with ARDS and the factors associated with bronchiectasis. To our knowledge, this is the first study to compare CT findings in the sequential follow-up with the reported traction bronchiectasis in COVID-19 survivors with ARDS.

## Materials and Methods

### Patients

**Figure 1:**
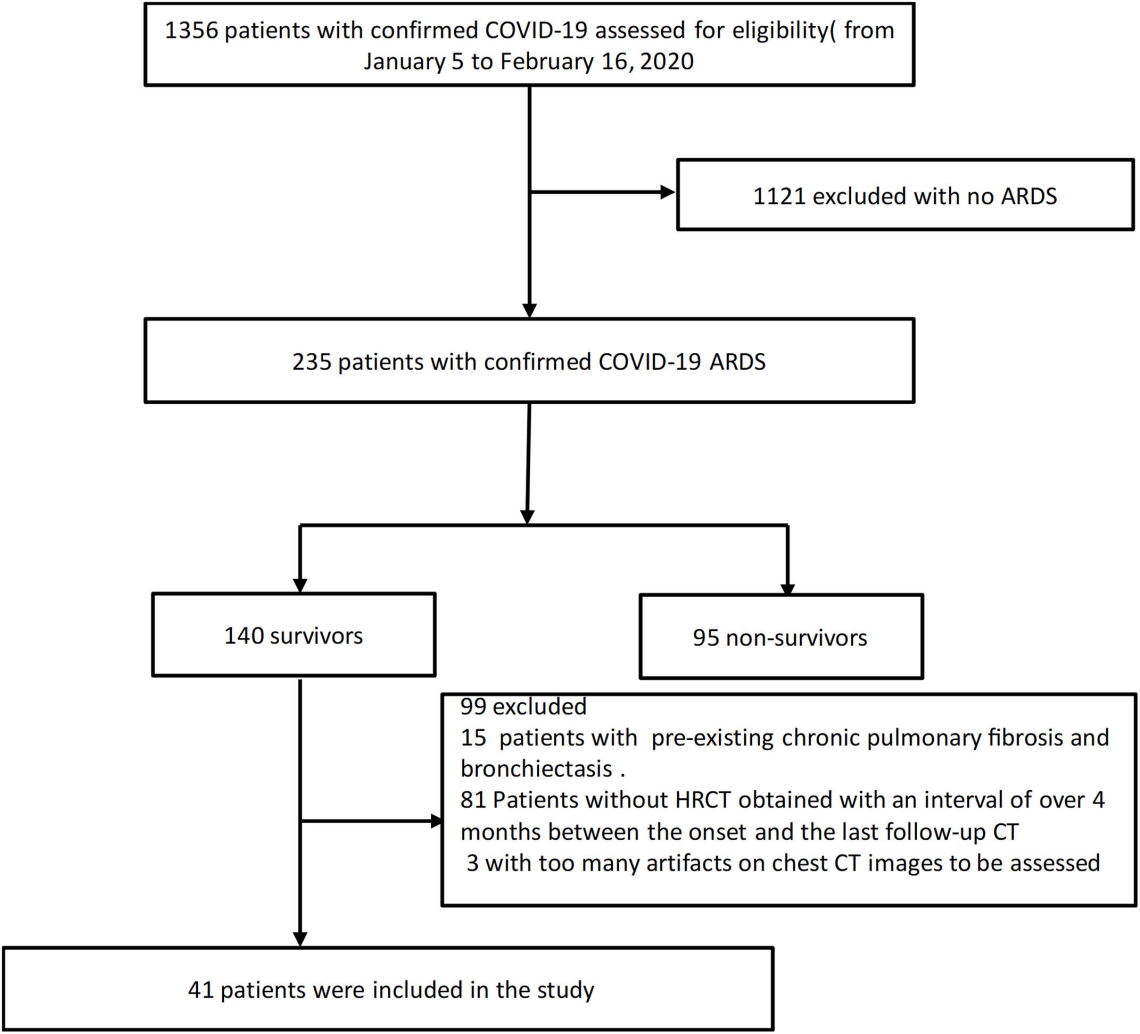
Study flow diagram.

The approval of the Institutional Review Board was obtained for this retrospective study (IRB ID: TJ-C0200108). Patient consent was waived because of the retrospective nature of the study and owing to emerging infectious diseases. Patients were admitted from January 5 to February 16, 2020.The inclusion criteria were as follows: (1) patients whose real-time reverse transcription polymerase chain reaction (RT-PCR) assay of throat swabs or nasopharyngeal swabs showed positive SARS-CoV-2 nucleic acid; (2) patients diagnosed as ARDS according to the Berlin definition (28); (3) survivor patients with an interval of over 4 months between the onset and the last follow-up CT scans. The exclusion criteria were described below: (1) patients with too many artifacts on chest CT images to be assessed; (2) patients previously diagnosed as bronchiectasis. The clinical data and the follow-up chest CT were monitored up to January 28 2021, the final date of follow-up. Forty-one COVID-19 survivors with ARDS were ultimately included in this study (Figure 1).

The demographics and clinical data (onset date of symptoms, diagnosis date of ARDS, days of hospitalization, oxygenation index, and comorbidities) of all the patients were collected and extracted by using the data collection forms.

### CT Examination

Computed tomography scanning was performed during inspiratory breath holding. All images were obtained with patients in the supine position using one of three CT systems (uCT 780, United Imaging, China; Optima 660, GE, America; Somatom Definition AS+, Siemens Healthineers, Germany). The imaging parameters were as follows: tube voltage, 80–120 kVp; automated tube current modulation (30–70 mAs); pitch, 0.984:1; slice thickness, 0.625–1.25 mm; matrix, 512 × 512; field of view, 350 × 350 mm, with the selection differences according to the machine types.

### CT Assessment

The CT images obtained in the first, second, third, and fourth weeks after the symptom onset and the last CT images were marked as CT_1_, CT_2_, CT_3_, CT_4_, and CT_L_. The CT image showing the most severe lung involvement was marked as CT_M_.

All CT images were assessed independently by two radiologists (YWL and QJH with 2 and 10 years of experience, respectively) and the final decisions reached by consensus were reported. According to the presence or absence of traction bronchiectasis on the CT image, the patients were divided into Group I (with traction bronchiectasis) and Group II (without traction bronchiectasis). The patients with traction bronchiectasis were further divided into Group IA (complete disappearance of traction bronchiectasis) and Group IB (incomplete disappearance of traction bronchiectasis).

The involvement extent of traction bronchiectasis on CT_1_, CT_2_, CT_3_, and CT_4_ images in Group IA and Group IB were evaluated. The lingula was considered to be a separate lobe. Each lung lobe was scored for the involvement extent based on the method of Reiff et al. (0 = none, 1 = one or partial bronchopulmonary segment involved, 2 = two or more bronchopulmonary segments involved) (29). The total involvement extent of bronchiectasis throughout the lungs was reflected by the sum of the scores of six lobes (bronchiectasis score, range: 0–12). The lobar distribution and location of traction bronchiectasis on CT_M_ images in Group IA and Group IB were evaluated. The location of bronchiectasis was categorized as central, peripheral, or mixed. The distinction between central and peripheral bronchial involvements was taken as a point midway between the hilum and the chest wall (30). The bronchiectasis scoring on CT_M_ and CT_L_ images of patients in Group IB was performed.

The evolution of lung involvement on CT images was analyzed by using three scoring methods. The first method was the overall lung “involvement score” (31, 32). Five lung lobes were separately assessed for the involvement extent (none (0%), minimal (1–25%), mild (26–50%), moderate (51–75%), and severe (76–100%), with a score of 0, 1, 2, 3, 4, respectively). The overall lung “involvement score” was calculated by summing five lobe scores (range: 0–20). The second method was “GGO/consolidation score”. The involvement extent of the lobes in ground-glass opacity (GGO) or consolidation on CT images was also scored according to the above method (range from 0 to 4). The third method was the overall “CT score” based on the classification by Ichikado et al. (33).

The presence of reticulation, fibrous stripes, pleural effusion, and lymph node enlargement on the last CT image was also evaluated. All CT findings were based on the recommendations in Fleischner Society terminologies (17).

### Statistical Analysis

All statistical analyses were performed using SPSS 21.0 software. The quantitative data were presented as mean ± standard deviation or median and compared by using Mann–Whitney U test. The qualitative data were presented as percentage (%) and analyzed with Fisher's exact test or Chi-square test. Wilcoxon signed rank test was used to compare the differences in the “involvement score” and “bronchiectasis score” between CT_M_ and CT_L_ in Group IB. *P* < 0.05 suggested that a difference was statistically significant.

## Results

A total of 41 COVID-19 survivors with ARDS were included in the study. The demographics, clinical data, and interval time are shown in Table 1; Supplementary Table 1. The median interval between the appearance of traction bronchiectasis and the onset of symptoms was 16 days, and the median interval between the appearance of traction bronchiectasis and the diagnosis of ARDS was 1 day.

**Table 1:** Baseline characteristics of all patients.

	**Total (N = 41)**	**Group I (N = 28)**	**Group II (N = 13)**	***P***
Sex^*^				0.503
Male	23 (56.1%)	17 (60.7%)	6 (46.2%)	
Female	18 (43.9%)	11 (39.3%)	7 (53.8%)	
**Age (years)**	63 [53–68]	64 [54–68]	62 [51–67]	0.537
**Oxygenation index /**mmHg	224 [159–232]	224 [164–232]	224 [153–251]	0.720
**Comorbidities**				
Hypertension	23 (56.1%)	15 (53.6%)	8 (61.5%)	0.447
Diabetes	10 (24%)	6 (21%)	4 (30.8%)	0.390
Heart disease	4 (9.8%)	2 (7.1%)	2 (15.4%)	0.377
Cerebrovascular disease	2 (4.9%)	2 (7.1%)	0 (0%)	0.461
Chronic lung disease	1 (2.4%)	1 (3.6%)	0 (0%)	0.683
Chronic liver disease	2 (4.9%)	1 (3.6%)	1 (7.7%)	0.539
Metabolic disease	4 (9.8%)	2 (7.1%)	2 (15.4%)	0.377
Hematopathy	1 (2.4%)	1 (3.6%)	0 (0%)	0.683
History of operation	12 (29.3%)	12 (42.9%)	0 (0%)	0.004
**Hospitalization (dates)**	38 [28–46]	41 [30–46]	30 [24–44]	0.144
Interval between the onset of symptoms and the diagnosis of ARDS (dates)	10 [9–13]	10 [9–13]	11 [8–14]	0.851
Interval between the onset of symptoms and the last CT scans (dates)	183 [150–215]	180 [145–202]	188 [165–279]	

*Except where indicated, the data are median, with IQR in the parentheses;*

* *The data are the number of patients, with percentage in the parentheses*.

**Figure 2:**
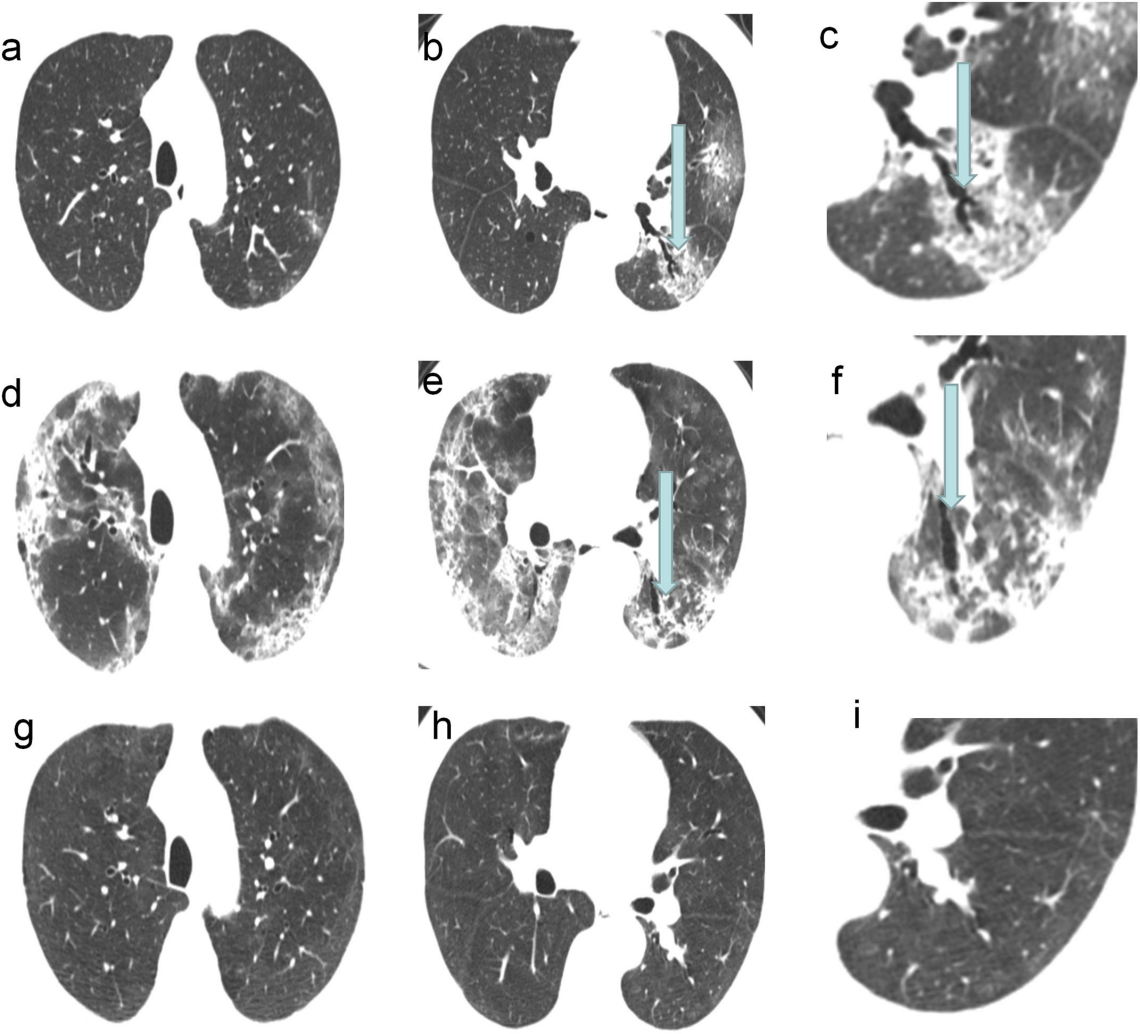
A 62-year-old COVID-19 patients with ARDS: CT scans showed the traction bronchiectasis disappeared completely. **(a–c)** CT images on the 7th day of the symptom onset showed traction bronchiectasis within area of GGO in the left lower lobe. **(d–f)** CT images on 19th day of the symptom onset showed the most severe involvement. **(g–i)** CT images on 133th day of the symptom onset showed the disappearance of traction bronchiectasis in the left lower lobe.

**Figure 3:**
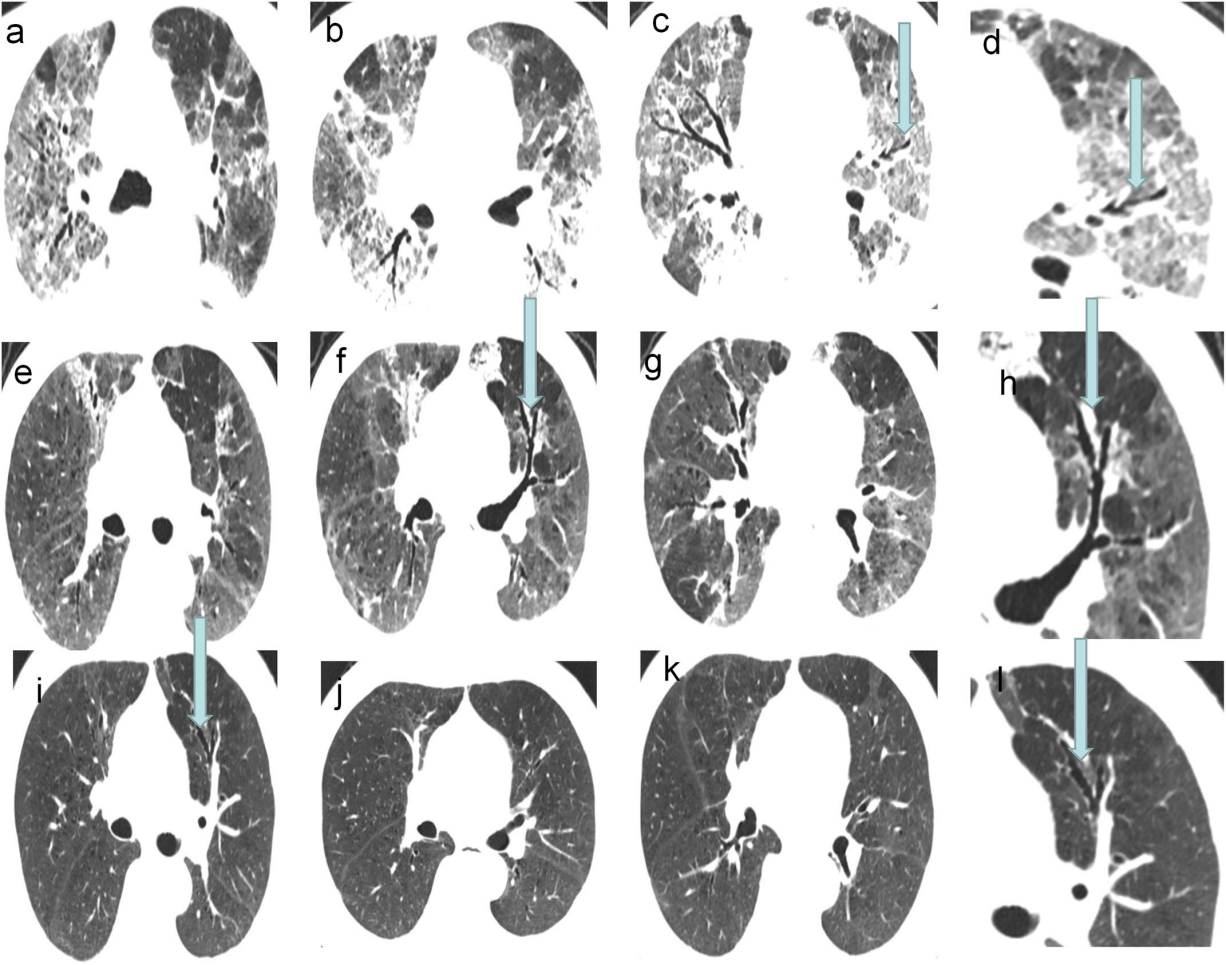
A 73-year-old COVID-19 patients with ARDS: CT scans showed the traction bronchiectasis relived and disappeared incompletely. **(a–d)** CT images on 24th day of the symptom onset. **(e–h)** CT images on 38th day of the symptom onset. **(i–l)** Last follow-up CT images, on 145th day of the symptom onset, showed the traction bronchiectasis was still present in the left upper lobe.

In the follow-up CT scans, 28 (68%) patients had traction bronchiectasis (Group I) and 13 (32%) patients had no traction bronchiectasis (Group II). Traction bronchiectasis disappeared completely in 21 (75%) patients (Group IA) (Figure 2) and was relieved, but did not disappear completely in seven (25%) patients (Group IB) (Figure 3). The interval between the disappearance of traction bronchiectasis and the onset of symptoms in Group IA was 135 [50-184] (median [IQR]) days.

There was no significant difference in the bronchiectasis score on CT_M_ between Group IA and Group IB (*P* > 0.05) (Supplementary Table 2). The distribution and location of bronchiectasis was predominantly wide (29%; 8/28) and peripheral (68%; 19/28). No significant difference in the bronchiectasis score within 4 weeks after the onset of symptoms was found between Group IA and Group IB (all *P* > 0.05) (Supplementary Table 3). Compared with CT_M_, the involvement extent of lung lobes in bronchiectasis on CT_L_ in Group IB was relieved (bronchiectasis score: 1.9 ± 1.1 vs. 6.6 ± 4.7; *P* = 0.002) (Table 2). Additionally, the total lung involvement in all patients was significantly improved (involvement score: 6.1 ± 4.4 vs. 13.9 ± 4.3; *P* < 0.001).

**Table 2:** Comparison of CT scores on CT_M_ and CT_L_ in patients with incomplete disappearance of traction bronchiectasis.

	**CT_**M**_**	**CT_**L**_**	***P***
Bronchiectasis score	6.6 ± 4.7 (1–12)	1.9 ± 1.1 (1–4)	0.002
Involvement score	13.9 ± 4.3 (8–20)	6.1 ± 4.4 (0–13)	0.000

*CT_M_ represents the CT images showing the most severe lung involvement; CT_L_ represents the last follow-up CT images; The data are mean ± standard deviation (SD), with range in the parentheses*.

At the second week after the onset of symptoms, the lung involvement on CT images in Group I was higher than that in Group II (lung involvement score: 14.7 ± 1.6 vs. 7.2 ± 1.7; *P* = 0.001) (Table 3). In addition, the CT images of patients in Group I showed much more GGO than those in Group II (*P* < 0.001). There was no significant difference in the CT score, GGO score, and consolidation score on CT_M_ between Group IA and Group IB (all *P* > 0.05) (Supplementary Table 2).

**Table 3:** CT scores in patients with and without traction bronchiectasis at different follow-up periods.

		**CT**_**1**_		**CT**_**2**_		**CT**_**3**_		**CT**_**4**_		**CT**_**L**_	
**Group**		**I**	**II**	**I**	**II**	**I**	**II**	**I**	**II**	**I**	**II**
**Number of CT scans**		**13**	**3**	**7**	**6**	**12**	**9**	**18**	**10**	**28**	**13**
Involvement score	mean ± SD (range)	7.3 ± 3.6 (1–14)	9.0 ± 3.9 (4–13)	14.7 ± 1.6 (12–17)	7.2 ± 1.7 (5–9)	10.3 ± 1.9 (7–13)	8.8 ± 3.9 (5–18)	11.1 ± 4.0 (6–20)	8.9 ± 3.7 (5–18)	4.5 ± 3.1 (0–13)	3.8 ± 2.8 (0–9)
	*P*	0.536		0.001		0.076		0.121		0.676	
GGO score	Right lung	1.4 ± 0.7 (1–3)	1.3 ± 1.0 (0–2)	3.0 ± 0.5 (2–4)	1.2 ± 0.4 (1–2)	1.9 ± 0.8 (1–3)	1.8 ± 1.0 (1–4)	2.1 ± 0.8 (1–4)	1.6 ± 0.5 (1–2)	1.0 ± 0.5 (0–2)	0.9 ± 0.6 (0–2)
	*P*	0.860		0.000		0.471		0.044		0.691	
	Left lung	1.3 ± 0.7 (0–3)	1.0 ± 0.8 (0–2)	2.3 ± 0.8 (1–3)	1.0 ± 0 (1–1)	1.8 ± 0.5 (1–2)	1.8 ± 1.0 (1–4)	1.9 ± 0.8 (1–4)	1.2 ± 0.4 (1–2)	0.9 ± 0.6 (0–3)	0.8 ± 0.7 (0–2)
	*P*	0.334		0.000		0.581		0.001		0.812	
Consolidation score	Right lung	1.1 ± 0.3 (1–2)	0.8 ± 1.0 (0–2)	1.7 ± 0.5 (1–2)	0.8 ± 0.4 (0–1)	1.5 ± 0.5 (1–2)	1.2 ± 0.4 (1–2)	1.3 ± 0.5 (1–2)	1.3 ± 0.8 (0–3)	0.4 ± 0.5 (0–1)	0.5 ± 0.5 (0–1)
	*P*	0.129		0.001		0.235		0.945		0.413	
	Left lung	0.9 ± 0.5 (0–2)	0.8 ± 1.0 (0–2)	1.3 ± 0.5 (1–2)	0.8 ± 0.4 (0–1)	1.3 ± 0.5 (1–2)	1.1 ± 0.3 (1–2)	1.3 ± 0.6 (0–2)	1.2 ± 0.8 (0–3)	0.4 ± 0.5 (0–1)	0.3 ± 0.5 (0–1)
	*P*	0.518		0.119		0.184		0.326		0.777	

*CT_1_, CT_2_, CT_3_, CT_4_ represents CT images of the first, second, third, and fourth weeks, respectively after the onset of symptoms; CT_L_ represents the last follow-up CT images; The data are mean ± standard deviation (SD), with range in the parentheses*.

The median interval between onset and last CT was 183 days (minimum 131 days and maximum 361 days). On the last CT images, 16 (76%) patients had fibrous stripes and three (14%) patients had reticulation in Group IA (*N* = 21) (Table 4). In Group IB (*N* = 7), five (71%) patients had fibrous stripes and four patients (57%) had reticulation. The last CT images of patients in Group IB were more likely to develop the reticulation than those in Group IA (*P* < 0.05).

**Table 4:** CT findings on the last follow-up CT images in patients with traction bronchiectasis.

**CT findings**	**Group IA (N = 21)**	**Group IB (N = 7)**	***P***
Reticulation	3 (14)	4 (57)	0.043
Fibrous stripe	16 (76)	5 (71)	1.000
Pleural effusion	0	1 (14)	0.250
Lymph node enlargement	3 (14)	3 (43)	0.144

*The data are the number of patients, with percentage in the parentheses*.

## Discussion

The presence of traction bronchiectasis is generally considered as the evidence of fibrosis (17, 34). Traction bronchiectasis may also occur in the acute phase of pneumonia or ARDS and affect the prognosis (11, 16, 35). It has been reported that traction bronchiectasis is found on the CT images of COVID-19 survivors with ARDS (8, 12, 13). However, the recent studies have shown that bronchiectasis is temporary and can be reversed by treatment in patients with severe infection and inflammation; thus it is called as pseudobronchiectasis (19–26), but it is rarely reported whether traction bronchiectasis is pseudobronchiectasis in patients with COVID-19 pneumonia. In the present study, we focused on investigating traction bronchiectasis in COVID-19 survivors with ARDS by comparing the findings from serial HRCT of lungs. Our study revealed that 28 (68%) COVID-19 patients with ARDS developed traction bronchiectasis, of which 21 achieved the complete resolution of traction bronchiectasis by treatment. This suggests that bronchiectasis in COVID-19 patients may be a dynamic process and associated with severe peribronchial inflammation.

In a study, 25/60 previously asymptomatic adult patients with pneumonia developed bronchiectasis during the acute phase, of which 20 (80%) were subsequently cured (36). At present, reversible bronchiectasis in patients with COVID-19 pneumonia is unknown. In our study, traction bronchiectasis was completely resolved in 21 (75%) of 28 COVID-19 patients with ARDS. This indicates that pseudobronchiectasis is present in COVID-19 patients. As shown by a case report of reversible bronchiectasis after mycoplasma pneumoniae pneumonia, an adult patient had complete resolution of bronchiectasis after 1 month of treatment (23). Maranatha et al. (27) reported a patient with severe COVID-19 and ARDS. CT images showed traction bronchiectasis in the lower posterior part of both lungs at the 30th day after the onset of symptoms and the disappearance of bronchiectasis at the 45th day. In this study, the median interval between the disappearance of traction bronchiectasis and the onset of initial symptoms was 135 days. Although it was different from two cases reported above, the number of cases in this study was relatively large, so it may have a better reference value. In general, patients with COVID-19 pneumonia are likely to develop traction bronchiectasis in the acute phase of ARDS, and such bronchiectasis is mostly reversible and may even be completely resolved by treatment. The definition and pathophysiology of pseudobronchiectasis has not been clearly illustrated (22, 25). Pseudobronchiectasis is found in the long-term follow-up of patients with non-specific interstitial pneumonia, which may be attributed to the collapse of surrounding peripheral lung parenchyma (21). Traction bronchiectasis in patients with COVID-19 under the acute phase of ARDS may be associated with severe peribronchial inflammation.

To further explore the factors associated with traction bronchiectasis in COVID-19 survivors with ARDS, we compared the lung involvement extent on CT images at 4 weeks after the onset of symptoms and on the last CT images. At the second week after the onset of symptoms, the CT images of patients with traction bronchiectasis showed the most severe lung involvement, and the mean involvement score was significantly higher than that of patients without traction bronchiectasis (14.7 ± 1.6 vs.7.2 ± 1.7; *P* = 0.001). The recent studies have suggested that the patients with COVID-19 pneumonia tend to progress most rapidly in the second week (37–39). Pan et al. (40) also found that the maximum lung involved peaked at ~10 days from the onset of initial symptoms. In the sequential follow-up CT scans of 41 patients, we found the lung involvement peak at the second week after the onset. The above findings have revealed that COVID-19 survivors with ARDS, who have more severe lung involvement on chest CT images, may be more likely to develop traction bronchiectasis.

Our study results showed that although traction bronchiectasis in seven patients was still present but was relieved on the last follow-up CT images, the lung involvement and total involvement extent of bronchiectasis on the last CT images were both significantly relieved as compared with CT images, showing the most severe lung involvement. On the last CT image of one patient, GGO and consolidation were completely absorbed. These results indicated that during the recovery period of patients with COVID-19 pneumonia, bronchiectasis was improved and was a dynamic process, which was associated with severe peribronchial inflammation. In our study, the patients with incomplete disappearance of traction bronchiectasis were more likely to have the reticulation on the last CT images than those with complete disappearance of traction bronchiectasis (57 vs. 14%; *P* < 0.05). Longer CT follow-up may be needed to track the evolution and outcome.

This study has several limitations. Firstly, it is a retrospective study. The available clinical data and image information are relatively limited. The potential deviations of the retrospective study design limit the interpretation of study results. Secondly, the number of cases included in this study is relatively small, and a larger sample size and longer follow-up are needed to better verify the conclusions. Furthermore, this study did not involve the pulmonary function and other clinical treatment and prognosis of patients.

In conclusion, COVID-19 survivors with ARDS developed pseudobronchiectasis, most of which achieved complete resolution. The findings of this study are interesting, for the presence of traction bronchiectasis is generally considered as the evidence of fibrosis (17, 34), and can pave the way for further investigation in this regard. For patients with incomplete disappearance on the last CT images, bronchiectasis was significantly relieved, but it still needs to be followed up whether bronchiectasis will further progress to fibrosis.

## Data Availability Statement

The original contributions presented in the study are included in the article/Supplementary Material, further inquiries can be directed to the corresponding author/s.

## Ethics Statement

The studies involving human participants were reviewed and approved by the Institutional Review Board (IRB) of Tongji Hospital, Tongji Medical College, and Huazhong University of Science and Technology (IRB ID: TJ-C0200108). Written informed consent for participation was not required for this study in accordance with the national legislation and the institutional requirements.

## Author Contributions

LX: guarantor of integrity of the entire study. YL, QH, and LX: manuscript editing and study concepts and design. YL and CC: statistical analysis. QH, YL, ZS, and MX: image collection. QH and YL: literature research. QH, YL, CC, ZS, YW, MX, HG, and LX: image analysis. All authors contributed to the article and approved the submitted version.

## Conflict of Interest

The authors declare that the research was conducted in the absence of any commercial or financial relationships that could be construed as a potential conflict of interest.

## Publisher's Note

All claims expressed in this article are solely those of the authors and do not necessarily represent those of their affiliated organizations, or those of the publisher, the editors and the reviewers. Any product that may be evaluated in this article, or claim that may be made by its manufacturer, is not guaranteed or endorsed by the publisher.
