# Supplementary material for: Reversible Bronchiectasis in COVID-19 Survivors With Acute Respiratory Distress Syndrome: Pseudobronchiectasis
Source: Front Med (Lausanne). 2021 Nov 30;8:739857. doi: 10.3389/fmed.2021.739857 (PMC8669592; doi:10.3389/fmed.2021.739857)
Supplement: Supplementary file 1 [file Data_Sheet_1.docx]

**Supplement Table 1: Baseline characteristics of 28 patients with traction bronchiectasis**

|  |  |  |  |
| --- | --- | --- | --- |
|  | **Group IA (N=21)** | **Group IB (N=7)** | ***P*** |
| **Sex*** |  |  |  |
| Male | 13(61.9%) | 4(57.1%) | 1.000 |
| Female | 8(38.1%) | 3(42.9%) |  |
| **Age, year** | 64[55-68] | 63[51-73] | 0.969 |
| **Oxygenation index**, mmHg | 224[177-232] | 232[156-251] | 0.865 |
| **Comorbidities** |  |  |  |
| Hypertension | 12(57.1%) | 3(42.9%) | 0.412 |
| Diabetes | 4(19.0%) | 2(28.6%) | 0.478 |
| Heart disease | 2(9.5%) | 0(0%) | 0.556 |
| Cerebrovascular disease | 2(9.5%) | 0(0%) | 0.556 |
| Chronic lung disease | 1(4.8%) | 0(0%) | 0.750 |
| Chronic liver disease | 1(4.8%) | 0(0%) | 0.750 |
| Metabolic disease | 2(9.5%) | 0(0%) | 0.556 |
| Hematopathy | 1(4.8%) | 0(0%) | 0.750 |
| History of operation | 7(33.3%) | 5(71.4%) | 0.093 |
| **Hospitalization (d)** | 43[30-46] | 35[24-51] | 0.631 |
| Interval between the onset of symptoms and the diagnosis of ARDS (d) | 10[9-12] | 11[8-19] | 0.572 |
| Interval between the onset of symptoms and the last CT scans (d) | 183[144-192] | 167[145-280] |  |
| Interval between the onset of symptoms and the appearance of bronchiectasis (d) | 20[10-24] | 15[8-28] | 0.969 |
| Interval between the diagnosis of ARDS and the appearance of bronchiectasis (d) | 0[0-11] | 1[-3-16] | 0.786 |
| Interval between the onset of symptoms and the disappearance of bronchiectasis (d) | 135[50-184] | / |  |

Note: Except where indicated, the data are median, with IQR in the parentheses; * the data are the number of patients, with percentage in the parentheses.

**Supplement Table 2: Evaluation results on CT_M_ in patients with traction bronchiectasis**

|  | **Total (N=28)** | **Group IA (N=21)** | **Group IB (N=7)** | ***P*** |
| --- | --- | --- | --- | --- |
| **Overall CT score** | 182±28 (130-243) | 179±28 (130-232) | 190±30 (150-243) | 0.412 |
| **GGO score** |  |  |  |  |
| Right lung | 2.4±0.8 (1-4) | 2.3±0.8 (1-4) | 2.4±1.0 (1-4) | 0.968 |
| Left lung | 2.0±0.7(1-4) | 1.9±0.6 (1-3) | 2.4±1.0 (1-4) | 0.143 |
| **Consolidation score** |  |  |  |  |
| Right lung | 1.4±0.5 (1-2) | 1.4±0.5 (1-2) | 1.4±0.5 (1-2) | 1.000 |
| Left lung | 1.4±0.5 (1-2) | 1.4±0.5 (1-2) | 1.4±0.5 (1-2) | 1.000 |
| **Bronchiectasis score** | 5.0±3.8 (0-12) | 4.5±3.5 (1-11) | 6.6±4.7 (1-12) | 0.228 |
| **Distribution of bronchiectasis*** |  |  |  |  |
| Predominant upper lobe | 8 (29) | 7 (33) | 1 (14) | 0.465 |
| Predominant lower lobe | 8 (29) | 5 (24) | 3 (43) |  |
| Upper and lower lobes | 4 (14) | 4 (19) | 0 |  |
| Widespread | 8 (29) | 5 (24) | 3 (43) |  |
| **Location of bronchiectasis*** |  |  |  |  |
| Central | 0 | 0 | 0 | 0.646 |
| Peripheral | 19 (68) | 15 (71) | 4 (57) |  |
| Mixed | 9 (32) | 6 (29) | 3 (43) |  |
|  |  |  |  |  |

Note: CT_M_ represents the CT images showing the most severe lung involvement; Except where indicated, the data are mean ±standard deviation (SD), with range in the parentheses; * the data are the number of patients, with percentage in the parentheses.

**Supplement Table 3: Bronchiectasis score in patients with traction bronchiectasis within 4 weeks after the onset of symptoms**

|  |  |  |  |  |  |
| --- | --- | --- | --- | --- | --- |
|  | **Group** | CT_1_ | CT_2_ | CT_3_ | CT_4_ |
| **Number of CT scans** | IA | 9 | 5 | 9 | 14 |
|  | IB | 3 | 2 | 3 | 4 |
| **Total** |  | 12 | 7 | 12 | 18 |
| **Bronchiectasis score** | IA | 1.1±1.7 (0-5) | 6.4±4.5 (1-10) | 2.6±3.5 (0-11) | 4.2±4.3 (0-12) |
|  | IB | 1.0±1.7 (0-3) | 1.5±0.7 (1-2) | 5.0±4.2 (2-11) | 7.8±4.7 (2-12) |
|  | *P* | 1.000 | 0.381 | 0.191 | 0.160 |
|  |  |  |  |  |  |

Note: CT_1_, CT_2_, CT_3_, CT_4_ represents CT images of the first, second, third, and fourth weeks after the onset of symptoms; the data are mean ±standard deviation (SD), with range in the parentheses.
